# Quality Assurance and Commissioning of an Infrared Marker-Based Patient Positioning System for Frameless Extracranial Stereotactic Radiotherapy

**Published:** 2007-12

**Authors:** Tejpal Gupta, Reena Phurailatpam, Mishra Ajay, Pai Rajeshri, Mohindra Pranshu, Chopra Supriya

**Affiliations:** 1 *Department of Radiation Oncology, Advanced Centre for Treatment Research & Education in Cancer (ACTREC), Tata Memorial Centre, Kharghar, Navi Mumbai, India;*; 2 *Department of Radiation Oncology, Tata Memorial Hospital, Mumbai, India*

**Keywords:** extracranial, infrared marker, patient positioning system, quality assurance, stereotactic radiotherapy

## Abstract

Rapid advancements in imaging technology have led to remarkable improvements in identification and localization of tumors, ushering the era of high-precision techniques in contemporary radiotherapy practice. However, uncertainties in patient set-up and organ motion during a course of fractionated radiotherapy can compromise precision of radiation therapy. Excellent accuracy has been achieved with invasive and non-invasive fixation systems for stereotactic radiotherapy. This report describes the commissioning procedure and Quality Assurance studies done to evaluate the accuracy of isocenter localization by an infrared marker-based positioning system (ExacTrac). The ExacTrac has two infrared cameras that emit and detect infrared rays from reflective markers and construct three-dimensional coordinates of each marker. It detects the difference of the actual isocenter position from the planned isocenter coordinates in three translational (lateral, longitudinal, vertical, or x,y,z axes) and three rotational axes (six degree of freedom). This study performed on a flat and static phantom shows excellent accuracy achieved by the ExacTrac system. The positioning accuracy of ExacTrac (± 1 mm translational displacement and ± 1° rotational errors) can be a valuable tool in implementing frameless extracranial stereotactic radiotherapy. Nevertheless, it needs to be further evaluated on patients with inherent motion and greater positional uncertainty before being adopted in clinical practice.

## INTRODUCTION

Rapid advancements in imaging technology have led to remarkable improvements in identification and localization of tumors. Contemporary software applications on modern treatment planning systems can produce highly conformal dose distributions around target volumes. The current generation of Linear Accelerators (LA) equipped with multileaf collimators, electronic portal imaging devices (EPID), and more recently in-room image guidance allows the implementation and verification of high-precision planning. All these have fuelled growth and ushered in the era of high-precision techniques in contemporary radiotherapy practice (1, 2, 3). However, uncertainties in patient set-up and internal organ motion (4) during a course of fractionated radiotherapy compromise the precision of radiation delivery.

Several groups have previously reported excellent accuracy (1-2 mm) achieved with invasive and non-invasive fixation systems (5-8) for stereotactic radiotherapy of intracranial targets making it a highly popular technique for brain tumors. Stereotactic radiotherapy of extracranial targets (9, 10) has not as yet achieved the same popularity possibly due to inherent movements of targets and greater positioning uncertainty and still continues to evolve. Improvement of patient alignment through the development of an accurate positioning system is a fundamental prerequisite for implementing high-precision techniques, including extracranial stereotactic radiotherapy (ESRT), in the clinic.

This report describes the commissioning procedure and Quality Assurance (QA) studies done to evaluate the accuracy of isocenter localization by an infrared marker-based positioning system (ExacTrac version 2.0, Brain-LAB, AG, Germany) for frameless ESRT.

## MATERIAL AND METHODS

### System configuration and Calibration

**Figure 1:**
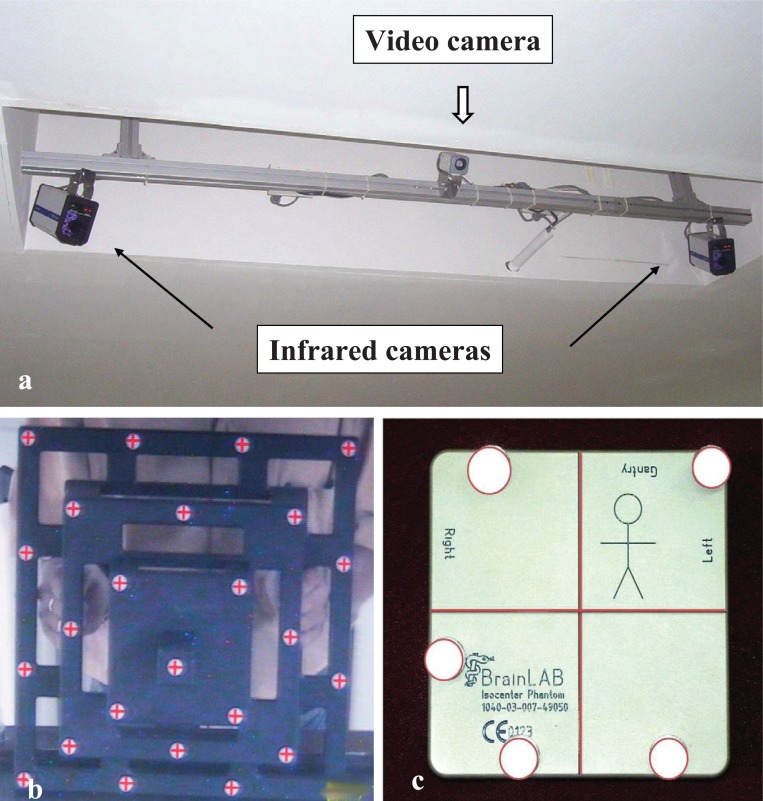
**(a)** Ceiling mounted set of ExacTrac infrared cameras (black arrows) and video camera (block arrow). Infrared cameras can receive and reflect infrared rays emitted from reflective markers; **(b)** Infrared and video camera calibration; **(c)** isocentre calibration.

The ExacTrac system consists of two infrared cameras (ICs), one video camera (VC), a set of infrared reflective markers (IRMs), networked computer workstation, in-room touch screen monitor, calibration accessories, and supporting software. The system needs to be calibrated at least once in a week to ensure maximum efficiency and reliability. There are three parts of calibration: Camera Calibration, Video Calibration and Isocentre Calibration (Figure 1).

**Camera Calibration:** A phantom having 25 reflective markers in fixed geometry is held in the field of view of the ICs, which must be focused towards the region of the isocentre of the LA (Figure 1a and 1b). The user confirms that 25 reflecting markers are seen by both ICs. If not, the phantom is moved until all the markers are seen. The analyzing software compares the detected three-dimensional (3D) co-ordinates of these markers with an internal reference look-up table and displays the variation. The position of the phantom is adjusted until the variation is within the specified tolerance limit and the calibration accepted.

**Video Calibration:** The next step is to check the correlation of the position of markers detected by both the infrared and video cameras. To check this, the VC displays an image of the phantom, with red crosses superimposed on the points at which the system determines the markers should be (Figure 1b). The user confirms that the image of the phantom obtained from the video camera has all of the crosses on the markers. Once all the positions match, the video calibration is complete. At this time, the phantom must be still for at least 3 seconds, and it is therefore recommended that it be placed on a table.

**Isocentre Calibration:** The isocenter calibration phantom is a 10 cm × 10 cm × 2 cm box with five IRMs on its anterior surface (Figure 1c). The engraved lines on top face and sides of the phantom are aligned precisely with the isocenter of the LA by collimator cross wire projections with gantry at 0°, 90° and 270° using the laser positioning system. Then the positions of the five IRMs are captured by the IC and a video image of the phantom acquired on the VC. The software recognizes the phantom, and superimposes a stored outline of the phantom on the image acquired by the video camera. If the user is satisfied that the contours match, the calibration is completed.

Following calibration, a comprehensive QA of the ExacTrac system was performed in two phases. In the first phase, the accuracy of the system in localizing the planned isocenter was evaluated. The second phase evaluated the accuracy of the digital values of the co-ordinates displayed by the ExacTrac system using EPID.

## EXPERIMENTS, OBSERVATIONS AND RESULTS

The Exactrac system has two ICs, which are both able to emit and detect infrared radiation. The reflective markers placed on the surface reflect the emitted beam to the cameras. The two infrared cameras are able to emit and detect infrared rays from the reflective markers and construct 3D coordinates of each marker. Both cameras need to detect one reflected beam each from a particular marker. This allows the system, which previously knows the relative separation and orientation of the two cameras, to calculate the position of the marker. At least four (preferably six) markers must be placed slightly asymmetrically on relatively immobile points on the patient’s surface or on the immobilization device in such a way that they are easily visible to both ICs.

**Figure 2:**
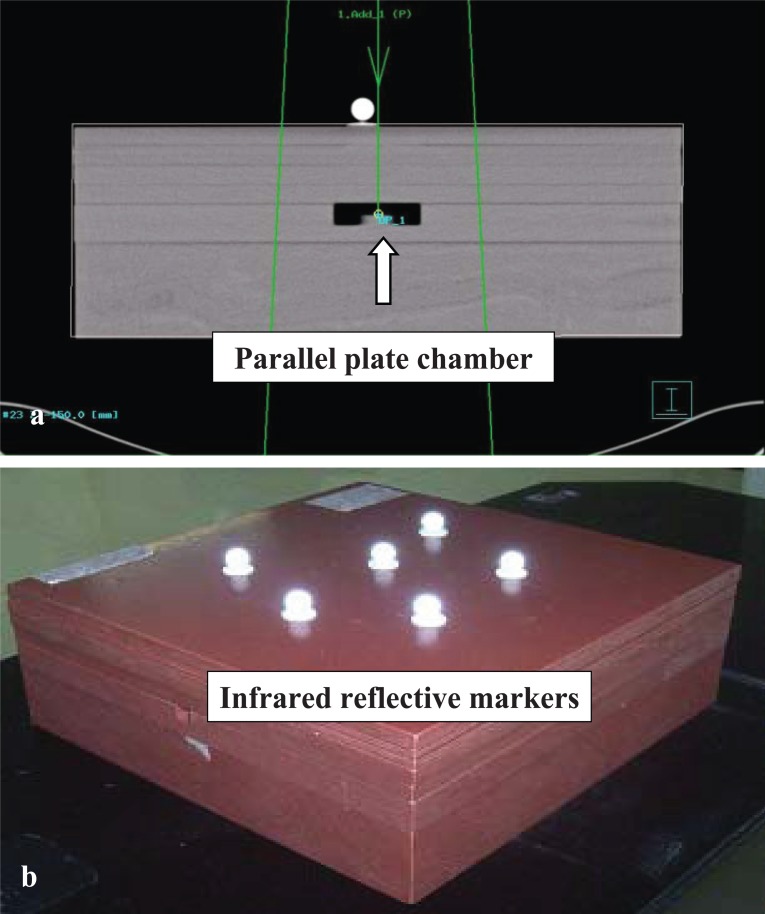
**(a)** Planning CT scan of virtual water phantom with parallel plate chamber (block arrow) at centre and infrared markers on surface; **(b)** Isocentre localization of solid water phantom using ExacTrac guidance.

A parallel plate chamber holder and two slabs of solid water phantom (5 cm thickness) were used. A radioopaque marker (1 mm diameter) was kept at the centre of the parallel plate chamber holder which was then placed the two solid water phantom slabs. Six IRMs were placed on the surface of the phantom asymmetrically such that they were visible to both the ICs. A planning computed tomography (CT) scan with 3 mm slice thickness was taken through the phantom, and virtual simulation was performed keeping the isocenter at the center of the radioopaque marker (Figure 2a). The projection of the isocenter was marked on the phantom surface. The plan was imported by the ExacTrac workstation, which automatically localizes the IRMs from the CT images and pre-defined isocenter position. The solid water phantom along with the 6 surface IRMs was oriented on the LA table in the same way as in the planning CT (Figure 2b). The ExacTrac monitor displayed the difference of the isocenter position from the planned isocenter coordinates in three translational (lateral, longitudinal, vertical, or x,y,z axes) and three rotational axes (six degree of freedom). Since the system was not equipped with automatic couch positioning software, the displacements were rectified by adjusting the phantom and table manually as per the ExacTrac display. When the isocenter was within the set tolerance (1 mm for translational and 1° for rotational axes), OK status was displayed on the ExacTrac monitor. The set-up Source-to-Surface Distance (SSD) on the phantom surface was verified with the optical distance indicator of the LA and was found to match exactly with the corresponding SSD from the treatment planning system. After removal of the upper slab of the solid water phantom, the optical crosswire and laser projections were found exactly at the centre of the 1 mm radioopaque marker placed at the centre of the parallel plate chamber holder.

The accuracy of the digital display of the ExacTrac system was verified using EPID. A set of orthogonal portal images were taken at gantry angles 0° and 270° keeping the phantom at the isocenter position. These were subsequently used as reference images. Translational errors (± 5 mm) in all three cardinal directions (x, y, and z axes), and rotational errors (± 2°) were introduced by table movements, and orthogonal portal images taken for each position of the table. The corresponding coordinates on the ExacTrac monitor (digital display coordinates) were also recorded. The displacements of the radioopaque marker at the centre of the chamber holder, relative to the reference orthogonal portal images were measured on the EPID workstation and compared with the actual errors introduced. The values of the digital scales on the ExacTrac monitor were found within ± 0.05 mm of the mechanical scale, suggesting excellent accuracy.

## DISCUSSION

Recent advances have produced tremendous growth in high-precision techniques in contemporary radiotherapy practice (1). Stereotactic radiotherapy of intracranial targets with millimetre accuracy (5-8) is reasonably well established in the clinic. ESRT however has not been able to achieve the same popularity possibly due to inherent movements of targets and greater positioning uncertainty. Improvement of patient alignment through the development of accurate positioning systems is a fundamental prerequisite for implementing high-precision techniques such as ESRT in routine clinical practice.

Traditionally, laser-based systems have been the preferred patient positioning method in fractionated radiotherapy (11), but its poor repositioning accuracy precludes usage for stereotactic irradiation. Several groups have been working with modern optico-electronic technology to develop novel patient positioning systems (12-19) for accurate delivery of radiation therapy. A review of the different commercially available image-guided radiation therapy systems is outside the scope of this discussion, and the reader is referred to an excellent contemporary review on this topic (20). In-room image-guidance systems are either gantry mounted or floor/ceiling mounted. The ExacTrac system is one such ceiling mounted infrared marker-based patient positioning and localization system that can achieve excellent accuracy for implementation of ESRT.

## CONCLUSION

This study performed on a flat and static phantom shows excellent accuracy achieved by the ExacTrac system. The positioning accuracy of ExacTrac (± 1 mm translational displacement and ± 1° rotational errors) can be a valuable tool in implementing frameless ESRT. Nevertheless, it needs to be further evaluated on patients with inherent random internal motion and greater positional uncertainty before being adopted in clinical practice.
